# Evaluation of SD BIOLINE *H. pylori* Ag rapid test against double ELISA with SD *H. pylori* Ag ELISA and EZ-STEP *H. pylori* Ag ELISA tests

**DOI:** 10.1186/s12907-018-0071-6

**Published:** 2018-03-05

**Authors:** Markos Negash, Afework Kassu, Bemnet Amare, Gizachew Yismaw, Beyene Moges

**Affiliations:** 1 Department of Immunology and Molecular Biology, School of Biomedical and Laboratory Sciences, College of Medicine and Health Sciences, University of Gondar, P.O.BOX 196, Gondar, Ethiopia; 2 Department of Medical Microbiology, School of Biomedical and Laboratory Sciences, College of Medicine and Health Sciences, University of Gondar, P.O.BOX 196, Gondar, Ethiopia; 3 Department of Biochemistry, School of Medicine, College of Medicine and Health Sciences, University of Gondar, P.O.BOX 196, Gondar, Ethiopia

**Keywords:** *Helicobacter pylori*, SD BIOLINE Ag rapid test, Stool antigen, Ethiopia

## Abstract

**Background:**

*Helicobacter pylori* antibody titters fall very slowly even after successful treatment. Therefore, tests detecting *H. pylori* antibody lack specificity and sensitivity. On the other hand, *H. pylori* stool antigen tests are reported as an alternative assay because of their reliability and simplicity. However, the comparative performance of *H. pylori* stool antigen tests for detecting the presence of the bacterium in clinical specimens in the study area is not assessed. Therefore, in this study we evaluated the performance of SD BIOLINE *H. pylori* Ag rapid test with reference to the commercially available EZ- STEP ELISA and SD BIOLINE *H. pylori* Ag ELISA tests.

**Methods:**

Stool samples were collected to analyse the diagnostic performance of SD BIOLINE *H. pylori* Ag rapid test kit using SD *H. pylori* Ag ELISA kit and EZ- STEP ELISA tests as a gold standard. Serum samples were also collected from each patient to test for the presence of *H. pylori* antibodies using dBest *H. pylori* Test Disk. Sensitivity, specificity, predictive values and kappa value are assessed. *P* values < 0.05 were taken statistically significant.

**Results:**

Stool and serum samples were collected from 201 dyspeptic patients and analysed. The sensitivity, specificity, positive and negative predictive values of the SD BIOLINE *H. pylori* Ag rapid test were: 95.6% (95% CI, 88.8–98.8), 92.5% (95%CI, 89–94.1%), 86.7% (95% CI, 80.5–89.6), and 97.6% (95% CI, 993.9–99.3) respectively.

**Conclusion:**

The performance of SD BIOLINE *H. pylori* Ag rapid test was better than the currently available antibody test in study area. Therefore, the SD BIOLINE Ag rapid stool test could replace and be used to diagnose active *H. pylori* infection before the commencement of therapy among dyspeptic patients.

## Background

*Helicobacter pylori,* a curved gram negative bacillus, has been etiologically associated with several pathogenic conditions of the stomach ranging from gastritis to gastric cancer [1–3]. Prevalence of *Helicobacter pylori* (*H. pylori*) infection varies based on several factors globally [4–6]. In developing countries more than 80% of the population is infected with *H. pylori* [7, 8].

According to the 2010 World Gastroenterology Organization report the prevalence of *H. pylori* in Ethiopia among the age groups 2–4 years, 6 years, and adults was 48%, 80% and > 95%, respectively [9]. It has been reported that dyspepsia is one of the commonest complaints in any Ethiopian outpatient department [10–12]. It is also reported to account 10% of hospital admissions in the country [13].

According to the American college of Gastroenterology, patients with un-investigated dyspepsia [14] can be diagnosed using different approaches [15–18]. Serology is a widely available and inexpensive test but with low diagnostic accuracy. On the other hand, the *H. pylori* stool antigen (HpSA) test has been put in the market as optional technique because of its reliability and simplicity. However, the comparative performance of HpSA tests for detecting presence of *H. pylori* in clinical specimens is not tested at the study area. Therefore, in this study, we determined the performance characteristics of the SD BIOLINE *H. pylori* Ag kit against the SD *H. pylori Ag* ELISA and commercial EZ-STEP *H. pylori Ag* ELISA tests by using stool specimen among dyspeptic patients attending the University of Gondar Hospital in Northwest Ethiopia, Gondar***.***

## Methods

### Study design, period and area

This facility based cross sectional study was conducted on clients with dyspepsia from February to March 2015 attending the medical outpatient department of the University of Gondar Hospital.

### Study participants

After informed consent was taken all dyspeptic patients with no prior eradication therapy were included in the current study.

### Sample collection and processing

Stool and blood specimens were collected from each patient for serologic tests. The blood was centrifuged until serum was separated and stored at -20^o^c. The stool specimens were also stored at -20^o^c until the lab tests were performed.

#### SD BIOLINE *H. pylori* Ag test

[Principle] The SD BIOLINE *H. pylori* Ag Rapid test kit result window has 2 pre-coated lines, “T” (Test Line) and “C” (Control Line). Both the Test Line and the Control Line in result window are not visible before applying any samples. The “T’” window coated with monoclonal anti-*H. pylori* will form a line after the addition of stool specimen (if there is *H. pylori* antigen). The Control window is used for procedural control and a line should always appear if the test procedure is performed correctly and the test reagents are working.

Stool specimens were subjected for the rapid test according to the manufacturer’s instruction (STANDARD DIAGNOSTIC, INC. Korea). In brief, after taking a portion of stool (about 50 mg) with sterile swab it was inserted into a specimen tube containing assay diluents to dissolve the sample. Next, 1 ml of sample diluents was added in a clean test tube. We waited for 5–10 min and used the upper layer for the test. Three drops (about 80 μl) were put into the sample wells of the test device. Test results were interpreted within 10–15 min. No interpretation was performed after 15 min.

A colour band will appear on the left section of the result window (control/“C” band and/or test/“T” band). The presence of only one band (“C” band) within the result window indicates a negative result while the presence of two colour bands (“T” band and “C” band) within the result window indicates a positive result. In case where the purple colour band was not visible within the result window (of the “C” window) after performing the test, the result was considered invalid and the specimen were re-tested using a new test kit.

#### SD *H. pylori* Ag ELISA

[Principle] Stools from patients are used as a source of sample for the determination of *H. pylori* antigen. Micro plates are coated with a cocktail of affinity purified monoclonal antibodies directed to the *H. pylori* antigens. In the 1st incubation, the solid phase is treated with the sample and simultaneously with a mixture of monoclonal antibodies to *H. pylori* conjugated with peroxidase (HRP). After washing out, in the 2nd incubation the bound enzyme specifically present on the solid phase generates an optical signal that is proportional to the amount of *H. pylori* antigens present in the sample.

The test was performed according to the manufacturer’s (STANDARD DIAGNOSTIC, INC., Republic of Korea (17099)) instruction. In brief, we prepared strip wells for negative control 3 wells, positive control 2 wells and sample wells. We pipette 100 μl of controls and patient’s stool samples to each well. Then we added 25 μl of Enzyme Conjugate (mixture of monoclonal antibodies to *H. pylori* and horse reddish peroxidase) to each well. The micro plates was covered with adhesive plate sealer and mixed well on vibrating mixer. The wells were incubated at 37 ± 1 degree centigrade for 60 min. The wells were washed 5 times with 350 μl of diluted washing solution and then mixed with 100 μl TBM Substrate A and 100 μl TBM Substrate B and incubated in the dark at room temperature for 10 min. A blue color will develop. Then 100 μl of Stopping Solution was added into each well in the same sequence and timing as the TMB addition. The blue color will change to yellow.

The absorbance of each well was read within 30 min at a wavelength of 450 nm with a reference filter of 620 nm. The individual values of the absorbance for the control were used to calculate the mean value if 0.005 ≤ A (neg.) ≤ 0.100 and A (pos.) ≥ 1.000. When one of the absorbance value of the negative controls was outside the specification, this value was neglected while both absorbance values of the positive control must comply with the specification. When these specifications were not met, the test was repeated. The mean absorbance of the negative controls was calculated to calculate the cut-off value by adding 0.100 [A (neg.) + 0.100 = cut-off value]. Based on the criteria of the test, the samples were classed as follows: A (sample) < cut-off≡*H. pylori* antigen negative; A (sample) ≥ cut-off≡*H. pylori* antigen positive. Samples with a test result greater or equal to the cut-off value were in duplicate. The test results were interpreted as follows:

Negative result: no detectable *H. pylori* antigen; Positive result: presence of detectable *H. pylori* antigen.

#### EZ-STEP *H. pylori* Ag ELISA

[Principle] the EZ-STEP *H. pylori* stool antigen test utilize polyclonal anti-*H. pylori* capture antibody adsorbed to micro wells. An aliquot of diluted patient samples is added to the micro well and incubated simultaneously with peroxidase conjugated polyclonal antibody, resulting in the *H. pylori* antigens being sandwiched between the solid phase and enzyme conjugate. After incubation at room temperature, the sample well is washed to remove unbound samples and enzyme labeled antibodies. Substrate is added and result can be read in 10 min. Any bound enzyme conjugate in the wells converts the colorless substrate to a blue color. Spectrophotometer determination will be done with the addition of stop solution. This commercially available *H. pylori* stool antigen test (DINONA, Inc., Seoul, Korea) was performed as per the manufacturer. In brief: 8 drops (about 400 μl) of sample diluents were added to a clean test tube. Using the sample collection stick provided, a portion of feces (about 0.1 g) was taken and inserted into the test tube containing Sample Diluents. The stick was sworn until the sample has been dissolved into the Sample Diluents.

Next, 3 drops (100 μl) of negative control in 2 wells, 3 drops (100 μl) of positive control in 2 wells, and 3 drops (100 μl) of samples prepared in each well were added using dropper provided. Then 3 drops (100 μl) of conjugate solution were added onto each negative control, positive control, and sample well. Then the plate was shaken on vibrating mixer for 15 s. The plate was sealed with the tape provided and incubated at room temperature for 60 min. Then plates were then washed with the diluted washing solution for 6 times (300 μl/well/cycle). Then 3 drops (100 μl) of substrate solution were added onto each well and shaken on vibrating mixer for 15 s and incubated at room temperature for 10 min in dark.

Next, 3 drops (100 μl) of Stopping Solution was added on wells before reading the absorbance for negative and positive controls using air blank. Absorbance was read in 15 min after adding the stopping solution at 450 nm with reference wavelength at 650 nm. The absorbance of positive control was all between 0.500 and 2.500 and the absorbance of two negative controls was between − 0.005 and 0.100. When the absorbance was − 0.005-0.000, then it may was calculated as 0.000. When it was out of the range, the test was repeated.

To calculate the mean absorbance of negative controls we used the following formula:

The mean absorbance of negative controls (NCx) = (N1 + N2)/2.

To calculate the mean absorbance of positive controls use the following formula:

The mean absorbance of positive controls (PCx) = (1.599 + 1.601)/2 = 1.600.

To calculate Cut off Value we used the following formula:

Cut off Value = NCx + 0.100.

Samples with absorbance higher than the cut off Value were considered positives while those lower than cut off Value were taken as negatives. When the result was interpreted as positive, test was repeated on 3 wells and a positive result in more than 1 well was interpreted as positive.

#### dBest *H. pylori* test disk

**[**Principle] This test contains a membrane strip, which is pre-coated with *H. pylori* capture antigen on test band region. The *H. pylori* antigen–colloid gold conjugate and serum sample moves along the membrane chromatographically to the test region (T) and forms a visible line as the antigen-antibody-antigen gold particle complex forms. This test device has a letter of T and C as “Test Line” and “Control Line” on the surface of the case. Both the Test Line and Control Line in result window are not visible before applying any samples. The Control Line is used for procedural control. Control line should always appear if the test procedure is performed properly and the test reagents of control line are working.

The dBest *H. pylori* Test Disk (Ameritech diagnostic reagent co ltd, Tongxiang, Zhejiang, China) test was performed according to the manufacturer’s instruction. In brief: The test disk was removed from the foil pouch and placed on a flat, dry surface. A drop (20–30 μl) of serum/plasma was applied on to the sample well. Then two drops of the buffer were added on the sample well. As the test began to work, purple colour was seen moving across the result window in the centre of the test disk. Test results were interpreted within ten minutes. The presence of two colour bands, ‘T’ and ‘C’, meant the test was positive while the presence of only one band (only on ‘C’) was interpreted as negative. If no band or single band only on ‘T’ was formed after 10 min, the result was considered invalid and the experiment was repeated.

### Statistical analysis

The data was cleaned and double entered on excel spread sheet and transported to SPSS version 20. Java Stat-two way contingency table analysis software (http://statpages.org/ctab2x2.html) was also used to calculate sensitivity, specificity, predictive and kappa values. In this study the results of SD BIOLINE *H. pylori* Ag test were compared with results of the reference methods (EZ- STEP ELISA and SD *H. pylori* Ag ELISA test). *P* values < 0.05 were taken statistically significant.

## Results

A total of 201 dyspeptic patients were included in the study of which 140 (69.7%) were males and the rest 60 (30.3%) were females. The age of the participants ranged from 7 to 85 years with a mean (±SD) of 29.5 years (±14.85). Stool samples from all participants were collected and analyzed by the three tests, namely: SD BIOLINE *H. pylori* Ag test, SD *H. pylori* Ag ELISA, and EZ-STEP *H. pylori* Ag ELISA. Accordingly, 75 (37.1%) of the participants were positive by the SD BIOLINE *H. pylori* Ag rapid test while 92 (45.8%) were positive by the SD *H. pylori* Ag ELISA. The EZ-STEP *H. pylori* Ag ELISA detected 81 (40.3%) of the samples as positives for *H. pylori* infection. On the other hand, 68 (33.8%) were positive using both ELISA tests (Table 1). The dBest *H. pylori* Test disk detected 143(71.1%) of the samples as positive.

**Table 1:** Serology results of the SD BIOLINE *H. pylori* Ag rapid test, SD *H. pylori* Ag ELISA test, and ZE-STEP *H. pylori* Ag ELISA at University of Gondar Hospital, 2015

		SD BIOLINE *H. pylori* Ag		
		Positive N (%)	Negative N (%)	Total N (%)
SD *H. pylori* Ag ELISA	Positive	72(96.0%)	20 (15.9%)	92 (45.8%)
	Negative	3 (4.0%)	106 (84.1%)	109 (54.2%)
EZ- STEP ELISA	Positive	65 (86.7%)	16 (12.7%)	81 (40.3%)
	Negative	10 (13.3%)	110 (87.3%)	120 (59.7%)
EZ-STEP + SD *H.pylori* ELISA	Positive	65 (86.7%)	3 (2.4%)	68 (33.8%)
	Negative	10 (13.3%)	123 (97.6%)	133 (66.2%)

*Ag* Antigen, *ELISA* Enzyme Linked Immunosorbent Assay, *N* number, *SD* Standard Diagnostics

The performance characteristics of the SD BIOLINE *H. pylori* Ag rapid test against the SD *H. pylori* Ag ELISA and EZ-STEP *H. pylori* Ag ELISA was summarized in Table 2. Only those samples which were positive/negative for both ELISA tests were considered as positive/negative and used for the calculation of the performance activity of the rapid test. The sensitivity, specificity, positive and negative predictive values of the SD BIOLINE *H. pylori* Ag rapid test were: 95.6% (95% CI, 88.8–98.8), 92.5% (95%CI, 89–94.1%), 86.7% (95% CI, 80.5–89.6), and 97.6% (95% CI, 993.9–99.3), respectively. The kappa value was 0.859 (95% CI, 0.759–0.906).

**Table 2:** Performance result of the SD BIOLINE *H. pylori* Ag rapid test and dBest *H. pylori* Test Disk rapid Antibody test using EZ- STEP ELISA and SD *H. pylori* Ag ELISA test as standard at University of Gondar Hospital, 2015

Type of test	Sensitivity %(95%CI)	Specificity %(95%CI)	PPV %(95%CI)	NPV %(95%CI)	Kappa value N(95%CI)	P value
Ag rapid test (SD BIOLINE)	95.6 (88.8–98.8)	92.5 (89–94.1)	86.7 (80.5–89.6)	97.6 (93.9–99.3)	0.859 (0.759–0.906)	< 0.001
Antibody rapid test (dBest)	75 (65.3–83.5)	30.8 (25.9–35.1)	35.7 (31.1–39.7)	70.7 (59.4–80.6)	0.046 (−0.069–0.147)	0.416

*Ag* Antigen, *CI* confidence interval, *ELISA* Enzyme Linked Immunosorbent Assay, *N* number, *NPV* negative predictive value, *PPV* positive predictive value, *SD* Standard Diagnostics

## Discussion

*H. pylori* infection can be diagnosed using either of the invasive and noninvasive approaches [17]. Among noninvasive techniques serology is the most widely used because it is cheap, simple and quick. However, it is unreliable to differentiate active and previous infection. Due to this its application especially to initiate and monitor eradication therapy has been in question. On the other hand, new noninvasive diagnostic test based on the detection of *H. pylori* stool antigen (HpSA) has been developed and made available to the market.

In the current study, the sensitivity of the SD BIOLINE *H. pylori* Ag rapid test was 95.6% (95%CI, 88.8–98.8%). The sensitivity of the test is generally comparable with other HpSA tests [19–22], higher than some [23–25] and a little bit lower than others [26–28]. In any way, the high sensitivity of this rapid test could improve the detection of active infections and enhance the confidence of physicians to prescribe eradication therapy.

The specificity of the test was also very high, 92.5% (95%CI, 89–94.1%). It was generally comparable with previous HpSA reports [20, 29], higher than some [25–27] and slightly lower than others [23, 24]. This high specificity could increase the reliability of the rapid test in identifying an active infection and easy discrimination from a previous exposure.

The positive predictive value (PPV) of the test in the current study was also high 86.7% (95%CI, 80.5–89.6%). It was comparable [30], higher [25, 26] and lower [23, 24] when compared to other studies. Likewise, the negative predictive value (NPV) was also very high, 97.6% (95%CI, 93.9–99.3%). It was comparable [19, 25], higher [23, 24] and lower [26, 27] when compared to previous studies. The high positive predictive value (PPV) and NPV values could show the higher accuracy of the rapid test. In addition, the kappa value of the target test was very high, 0.859 (95%CI, 0759–0.906), which shows an excellent agreement between the rapid test and the reference standard used.

While a performance characteristic is typically compared against a gold standard test, we evaluated the SD BIOLINE *H. pylori* Ag rapid test kit against an already commercialized antigen tests, EZ-STEP *H. pylori* Ag test (DINONA, Inc., Korea) and SD *H. pylori* ELISA Ag tests (double ELISA) because of the lack of gold standard test in the study area. In addition, it is important to make note that the various tests compared above are based on their use of HpSA. They have also used different techniques as gold standards.

The sensitivity of the antibody test was 75% (95% CI, 65.3–83.5) which was much lower than the sensitivity of the SD BIOLINE *H. pylori* Ag test. Similarly, the specificity of the antibody test was also much lower than for the SD BIOLINE *H. pylori* Ag test, 30.8% (95% CI, 25.9–35.1%). *H. pylori* immunoglobulin G (IgG) serology detects an immune response, which could represent either a current infection or a previous exposure. Using such antibody tests may then predispose to an overuse of this drugs which could have a negative economic impact, increased risk of drug resistance, and exposure to unnecessary drug adverse effects.

## Conclusion

The SD BIOLINE *H. pylori* Ag rapid test has a much better sensitivity, specificity and predictive values compared to the currently available antibody test in the market, in Ethiopia. Therefore, the SD BIOLINE *H. pylori* Ag rapid stool test could be used to diagnose active *H. pylori* infection before the commencement of eradication therapy. However, further studies are required on how to use this HpSA rapid test for monitoring of therapeutic response or test of cure.

## Abbreviations

AUC: Area under curve
CIs: Confidence intervals
*H. pylori*: *Helicobacter pylori*
HpSA: *H. pylori* stool antigen
IgG: Immunoglobulin G
IRB: Institutional Review Board
NPV: Negative predictive value
PPV: Positive predictive value
